# Selenium-Induced Enhancement in Growth and Rhizosphere Soil Methane Oxidation of Prickly Pear

**DOI:** 10.3390/plants13060749

**Published:** 2024-03-07

**Authors:** Yiming Wang, Xuechong Xie, Huijie Chen, Kai Zhang, Benliang Zhao, Rongliang Qiu

**Affiliations:** 1 Guangdong Laboratory for Lingnan Modern Agriculture, College of Natural Resources and Environment, South China Agricultural University, Guangzhou 510642, China; wym568354@stu.scau.edu.cn (Y.W.); xxc0123@stu.scau.edu.cn (X.X.); hjchen2024@163.com (H.C.); zkdr1694917025@163.com (K.Z.); qiurl@scau.edu.cn (R.Q.); 2 Guangdong Provincial Key Laboratory of Agricultural & Rural Pollution Abatement and Environmental Safety, Guangzhou 510642, China

**Keywords:** selenium, *Roxburgh roxburghii*, physiological response, selenium translocation, methane oxidation

## Abstract

As an essential element for plants, animals, and humans, selenium (Se) has been shown to participate in microbial methane oxidation. We studied the growth response and rhizosphere methane oxidation of an economic crop (prickly pear, *Rosa roxburghii* Tratt) through three treatments (Se0.6 mg/kg, Se2.0 mg/kg, and Se10 mg/kg) and a control (Se0 mg/kg) in a two-month pot experiment. The results showed that the height, total biomass, root biomass, and leaf biomass of prickly pear were significantly increased in the Se0.6 and Se2.0 treatments. The root-to-shoot ratio of prickly pear reached a maximum value in the Se2 treatment. The leaf carotenoid contents significantly increased in the three treatments. Antioxidant activities significantly increased in the Se0.6 and Se2 treatments. Low Se contents (0.6, 2 mg/kg) promoted root growth, including dry weight, length, surface area, volume, and root activity. There was a significant linear relationship between root and aboveground Se contents. The Se translocation factor increased as the soil Se content increased, ranging from 0.173 to 0.288. The application of Se can improve the state of rhizosphere soil’s organic C and soil nutrients (N, P, and K). Se significantly promoted the methane oxidation rate in rhizosphere soils, and the Se10 treatment showed the highest methane oxidation rate. The soil Se gradients led to differentiation in the growth, rhizosphere soil properties, and methane oxidation capacity of prickly pear. The root Se content and Se translocation factor were significantly positively correlated with the methane oxidation rate. Prickly pear can accumulate Se when grown in Se-enriched soil. The 2 mg/kg Se soil treatment enhanced growth and methane oxidation in the rhizosphere soil of prickly pear.

## 1. Introduction

Selenium (Se) is an essential trace element with indispensable biological functions at the cellular and molecular levels. Se deficiency in soils is widespread worldwide, and the global mean Se content in soil is around 0.4 mg/kg, ranging from 0.1 to 2.0 mg/kg [1,2]. Se deficiency in soils is one of the leading causes of Se deficiency in plants and humans. Se deficiency has been found to be the underlying cause of several metabolic diseases in humans, such as cardiovascular diseases, osteochondrosis, low immune function, cancer, etc. [3,4]. Se supplementation has been demonstrated to enhance human immunity, boost anti-aging processes, and reduce the incidence of cancer [4]. As an effective way to increase Se content in humans, dietary uptake is a major source of Se supplements [5]. Studies have shown that crops grown in Se-rich soils tend to have relatively high Se levels [5]. The utilization of Se-rich soils is particularly necessary in the production of Se-rich foods. The efficient use of Se-rich soils has supported the Se-rich food industry in China, although China is one of more than 40 countries with Se-deficient soils [6]. Se-rich agricultural products have provided a variety of approaches to human Se supplementation in China, such as Se-rich tea (*Camellia sinensis* (L.) O. Kuntze) [7], Se-rich cabbage (*Brassica oleracea* L.) [8], Se-rich citrus (*Citrus sinensis* L. Osbeck) [9], Se-rich wheat (*Triticum aestivum* L.) [10], etc. Furthermore, rational application of Se in soil can improve the yield, quality, and Se content of cultivated crops. The addition of 2 mg/kg Se to soil increased the rice grain biomass by 39.3% [11]. The application of 37.5 g/ha Se fertilizer to soil increased wheat yields by 31.4% [12]. In addition, low-dose Se (0.5 mg/L) increased the yield of grapes, and high-dose Se (1.25 mg/L) increased the Se and nutrient contents, including total phenolics, flavonoids, and antioxidant capacity, in grapevine [13].

Prickly pear (*Rosa roxburghii* Tratt) is a perennial shrub that is mainly cultivated in the Yunnan-Guizhou Plateau and the western Sichuan Plateau in China [14]. Studies have shown that the prickly pear fruit is rich in vitamins, inorganic salts, polysaccharides, superoxide dismutase, and diverse amino acids, and the Se content of prickly pear exceeds 11 μg/100 g, meeting the standard of Se-rich agricultural products [15,16]. Prickly pear leaves contain bioactive ingredients such as polyphenols, polysaccharides, flavonoids, vitamin C, amino acids, etc. Prickly pear leaves have been processed extensively for prickly pear tea due to their unique tea aroma and health effects in the market [17]. By 2022, the cultivation area of prickly pear had reached 140,000 ha, and the economic value had exceeded USD 2.1 billion [18].

Methane is the second most important anthropogenic greenhouse gas after carbon dioxide. Its production and consumption have aroused widespread concern with respect to global climate change [19]. Studies have shown that soil methanotrophs contribute 10–20% to global methane consumption, playing a key role in methane balance [20]. The microhabitat of plants’ rhizosphere is closely linked to the methane oxidation process in soil, as methanotrophs are usually concentrated in the rhizosphere [21,22]. Oxygen is diffused from the roots into the plants’ rhizosphere and the adjacent soil through radial oxygen loss, creating a favorable microhabitat for aerobic methane oxidation [23].

The application of Se can improve photosynthesis, antagonize the toxicity of heavy metals, and enhance plants’ stress tolerance [3,9,24]. Appropriate amounts of Se can promote root growth and metabolic activities [25]. Recent studies have reported that methane oxidation consortia possess selenate-reducing activity [26,27,28]. Se may participate in the methane oxidation process in the rhizosphere microhabitat, due to Se metabolism and methane oxidation. We assumed that Se in soil could affect the methane oxidation process within the rhizosphere. Therefore, the cultivation of prickly pear in Se-rich soils may exert a potential ecological impact on the regulation of soil methane emissions.

Se enrichment characteristics in prickly pear fruit have been studied previously [16]. However, limited studies have examined Se’s uptake and translocation in prickly pear plants. The methane oxidation responses of prickly pear’s rhizosphere in Se-rich soils are not clear. We studied the growth, physiological response, and methane oxidation in the rhizosphere of prickly pear planted in Se-rich soils, aiming to explore the following questions: (1) Do gradients of soil Se cause differential growth of prickly pear? (2) Does prickly pear accumulate Se when grown in Se-rich soil? (3) Is methane oxidation enhanced in the prickly pear rhizosphere in Se-rich soils? This study contributes to the further understanding of Se uptake characteristics and the ecological effects of prickly pear cultivation in Se-rich soils.

## 2. Materials and Methods

### 2.1. Soil and Plant Materials

The soil was collected from the upper layer (0–20 cm) of Bazhai Farm in Bijie, Guizhou Province, China (27.45° N, 105.39° E). The soil samples were carefully cleaned of weeds, roots, and stones before being air-dried. The soil used for the prickly pear pots was sifted through a 2 mm mesh, and then, its basic physicochemical characteristics were determined (Supplementary Table S1).

The prickly pear seedlings were sourced from Bazhai Farm, located in the city of Bijie, Guizhou Province, China. The potted prickly pear experiment was carried out on the farm of South China Agricultural University (112.96° N, 23.43° E). The region has a subtropical monsoon climate with temperatures ranging from 21.5 to 22.2 °C, annual precipitation of 1696.5 mm, and average relative humidity of 77%.

Selenite is a major form of Se in acidic soil, and its toxicity is lower than that of selenate [29]. We used sodium selenite (AR, Aladdin Reagent Co., LTD., Shanghai, China) as the Se source in the pot experiment. The Se standard solution was 100 μg/mL (Beijing Yihua Tongbiao Technology Co., LTD., Beijing, China). Nitric acid, hydrochloric acid, and perchloric acid were of GR grade (Guangzhou Chemical Reagent Factory, Guangzhou, China). Potassium borohydride (Zixing Chemical Company, Guangzhou, China), thiourea (Aladdin Reagent Co., LTD., Shanghai, China), and ascorbic acid (Aladdin Reagent Co., LTD., Shanghai, China) were of AR grade.

### 2.2. Pot Experiment

Three Se treatments and one control were used in the pot experiment, and the amounts of Se applied were 0 mg/kg (CK), 0.6 mg/kg (Se0.6), 2 mg/kg (Se2), and 10 mg/kg (Se10). Before the pot experiment, sodium selenite was dissolved in pure water. The soils with selenite were stirred and blended evenly after the selenite solution was sprayed into the soil [30]. Then, the soil with experimental gradients was grouped into pots, with five replicates for each gradient. The pots were arranged randomly, and pure water was added to each pot to maintain the relative water content in the soil at 70%. Pure water with pH 6.8, conductivity 0 μs/cm, and TOC < 10 ppb was obtained using a water purification instrument (LT-RY10, Chongqing Loncent Technology Co., Ltd., Chongqing, China). After 30 days of soil Se equilibrium, N, P_2_O_5_, and K_2_O were applied at 150 mg/kg, 100 mg/kg, and 100 mg/kg, respectively. The final soil Se content of the CK, Se0.6, Se2, and Se10 treatments was 0.01 ± 0.004 mg/kg, 0.58 ± 0.011 mg/kg, 1.96 ± 0.022 mg/kg, and 9.92 ± 0.054 mg/kg, respectively. Prickly pear seedlings in a similar growth state, with a height of 10 ± 1 cm, were selected for transplantation in March 2023. Each pot had two seedlings. The same daily management for prickly pear was performed for the treatments and the control. Water was supplied every 3 days, and the relative water content in the soil was maintained at 70%. Weeds and pests were checked daily and manually removed, ensuring the growth of prickly pear and avoiding the possible disturbance of herbicides or pesticides. The experiment went on for two months.

### 2.3. Determination of the Growth Characteristics of Prickly Pear

The height of the prickly pear plants was recorded as the distance from the base to the top of the seedling using a straight ruler at the end of the pot experiment [31]. The stem diameter was measured at the base of the prickly pear seedling using an electronic caliper (DL91150, Deli Group Co., Ltd., Foshan, China) [31]. Both prickly pear plants seedlings were removed from the pot and cleaned with pure water; one was dried for the determination of biomass, and the other was placed in liquid nitrogen with dry ice for the determination of photosynthetic pigments, antioxidant enzyme activity, MDA, and soluble protein content.

The prickly pear plants were dried at 105 °C for 30 min in an oven (DHG-9070A, Shanghai Yiheng Technology Instrument Co., Ltd., Shanghai, China). Subsequently, the plant samples were dried at 80 °C until they reached a constant weight [32], and the biomass of each part of the prickly pear was weighed using an electronic balance (LC-FA1204, Licheng S&T Co., Ltd., Shanghai, China). The plant samples were ground through a 100-mesh sieve and stored in a dryer for further measurement.

All of the newly grown, fully unfolded fresh leaves of the prickly pears were preserved in liquid nitrogen for the determination of the following indicators: The photosynthetic pigments of prickly pear leaves, including chlorophyll a, chlorophyll b, and carotenoids, were determined by ethanol extraction using an ultraviolet spectrophotometer (UV-1600PC, Juneng Instrument Equipment Co., Ltd., Hangzhou, China) [33]. Fresh leaves (0.1 g) of prickly pear were added with 1 mL of the extraction solution (W: V = 1:10). The solutions were homogenized in an ice bath and centrifuged at 8000× *g* and 4 °C for 10 min. The supernatants were used to measure the catalase (CAT), peroxidase (POD), and superoxide dismutase (SOD) activities and malondialdehyde (MDA) contents. CAT activity was measured by the oxidation rate of H_2_O_2_ at 240 nm wavelength, and 1 μmol of H_2_O_2_ degradation per g of tissue per minute was defined as a unit of enzyme activity [34], using the catalase activity assay kit (AKAO003-2C, Beijing Boxbio S&T Co., Ltd., Beijing, China). POD activity was detected by the amount of 4-o-methoxyphenol produced by H_2_O_2_ oxidation of guaiacol [35], using the superoxide dismutase activity assay kit (AKAO001C, Beijing Boxbio S&T Co., Ltd.), where a change of 0.01 in absorbance per minute corresponds to a unit of POD activity. SOD activity was measured by monitoring the photoreduction of nitro blue tetrazolium [36] using the peroxidase activity assay kit (AKAO005M, Beijing Boxbio S&T Co., Ltd.), and one unit of SOD activity was defined as 50% inhibition of nitro blue tetrazolium reduction at 560 nm. MDA was measured using an assay of thiobarbituric-acid-reactive substances, which was performed to determine the extent of lipid peroxidation [37] using the malondialdehyde content assay kit (AKFA013C, Beijing Boxbio S&T Co., Ltd.). The soluble protein content was determined using the Coomassie brilliant blue method [38].

The roots were cleaned 3–5 times with pure water, for one minute each time. The entire root of the prickly pear was scanned using a root scanner (LD-WinRHIZO, Lind Intelligent Tech Co., Ltd., Jinan, China). The morphological parameters of root length, root surface area, and root volume were determined through WinRHIZO analysis [39]. The fresh root (0.2 g) was collected to measure the triphenyl tetrazolium chloride reduction capability of root dehydrogenase. Root activity was measured at 485 nm [40].

### 2.4. Determination of Rhizosphere Soil Properties

The pH and conductivity of the soil were measured using a multifunctional water quality detector (SX713, LABSEN, Shanghai, China). The pH was determined for a soil–water ratio of 1:2.5, and the extraction solution was a 1 mol/L KCl solution [41]. The soil conductivity was determined at a soil–water ratio of 1:5, and the extraction solution was pure water [41]. The soil organic matter was determined through dichromate heating oxidation, and the organic carbon was calculated [42]. The total N content was determined by means of the Kjeldahl method [41]. The total P content in the soil was determined by means of the sodium hydroxide melt-molybdenum-antimony resistance colorimetric method [41]. The total K content was determined using sodium hydroxide melt-flame spectrophotometry [41]. The alkali-dissolved N content was determined by means of the alkali diffusion method [42]. The soil’s available P was extracted using NH_4_F and determined by means of molybdenum–antimony colorimetry [41]. The available K content was extracted using NH_4_OAc and subsequently measured via flame photometry [43]. The change ratio of organic carbon, alkali-dissolved N, available P, and available K was calculated according to the final contents:

$$CR=(C_{f}-C_{b})/C_{b}$$

where *CR* is the change ratio (%) of organic carbon, alkali-dissolved N, available P, or available K; *C_f_* is the final soil organic carbon, alkali-dissolved N, available P, or available K content (mg/kg); and *C_b_* is the basic soil organic carbon, alkali-dissolved N, available P, or available K content (mg/kg).

A mixture of nitric acid and perchloric acid was added to the digestion tubes with the plant or soil samples (0.1 ± 0.0001 g) before digestion in the graphite furnace digester at 180 °C (Labtech Digiblock ED36, LabTech Inc., Boston, MA, USA). After cooling, hydrochloric acid was added to continue the digestion. The resultant solution was filtered for further measurement. The Se contents in the soil and parts of prickly pear were determined by means of atomic fluorescence spectrometry (AFS-12002, Jitian, Beijing, China) [44].

The translocation factor (TF) was used to characterize the ability of prickly pear roots to take up Se and transport it to the aboveground parts [45]:

$$TF=C_{shoot}/C_{root}$$

where *C_shoot_* and *C_root_* are the Se concentration (mg/kg) in the aboveground and underground parts of the prickly pear, respectively.

### 2.5. Determination of the Methane Oxidation Rate in Rhizosphere Soil

After being removed from the pot, the prickly pear roots were gently shaken, and rhizosphere soil attached to the roots was used to measure the methane oxidation capacity. After sifting through a 2 mm mesh, soil samples (10 g) from the treatments and control were placed in a 150 mL serum bottle. The relative soil water content in the serum bottles was adjusted to 70%. After washing with argon, 140 mL of standard argon was injected into the bottle. Following extraction of 10 mL of standard argon, pure methane (10 mL) was added to the bottle. The initial methane concentration was measured (0 h). The serum bottle was incubated at 25 ± 1 °C for 96 h, and the methane concentration was measured at 12 h, 24 h, 48 h, 72 h, and 96 h; the relative methane reduction was calculated based on two sequential adjacent times. The soil methane oxidation rate (MOR) was calculated according to the difference in methane concentration between the two measurements, using Equation (1) [46]:

(1) $$MOR=(C_{0}-C_{1})\times V\times \rho /(t\times m)$$

where *MOR* is the methane oxidation rate (nmol/g/h), *C*_0_ and *C*_1_ are the initial and final concentrations (µL/L) of methane in the serum bottle, respectively, *V* is the volume of air in the bottle (140 mL), *ρ* is the gas density of methane under standard conditions (0.717 g/L), *t* is the incubation duration (h), and *m* is the dry soil mass (g). The methane concentrations in the gas samples were determined using a gas chromatograph with a hydrogen-ion flame detector (GC-7890A, Agilent Technologies, Inc., Santa Clara, CA, USA) [47].

## 3. Data Analysis

Data were sorted using Excel software (Excel 2016, Microsoft, Washington, DC, USA). Data normality testing, variance homogeneity testing, and one-way analysis of variance were performed using SPSS software (version 26.0, IBM, Armonk, NY, USA). Multiple comparisons were performed using the least significant difference (LSD) test for significant differences (*p* < 0.05 or 0.01). The relationships between rhizosphere soil properties and root characteristics were analyzed through correlation analysis using the R package ‘stats’ [48]. The rhizosphere soil properties and root characteristics were subjected to principal component analysis using the R packages ‘FactoMineR’ and ‘factoextra’ [48,49,50]. Data plots were constructed using Origin 2021b software (OriginLab, Northampton, MA, USA, 2021).

## 4. Results

### 4.1. Effect of Se on Prickly Pear Growth

The growth of prickly pear was significantly affected by different soil Se contents (Table 1). The total biomass of prickly pear increased significantly in the three Se treatments compared to CK (*p* < 0.05), and the maximum biomass was found in the Se2 treatment. Changes in the root and leaf biomass of prickly pear showed an increasing trend followed by a decrease with the increase in Se content in the soil. The root biomass in the Se0.6 and Se2 treatments was significantly higher than that in CK (*p* < 0.05) (Table 1). The leaf biomass in all three Se treatments was higher than in CK. The stem biomass of prickly pear was significantly enhanced in the three Se treatments compared to CK (*p* < 0.05), while no significant differences existed between treatments. The root/shoot values of prickly pear in the Se2 treatment significantly increased compared to CK (*p* < 0.05). With the increase in soil Se content, the prickly pear height first increased and then decreased (Supplementary Figure S1). The height of prickly pear plants in the Se2 and Se0.6 treatments was higher than in CK and the Se10 treatment (*p* < 0.05). No significant differences in the stem thickness of prickly pear were observed in the Se treatments.

**Figure 1:**
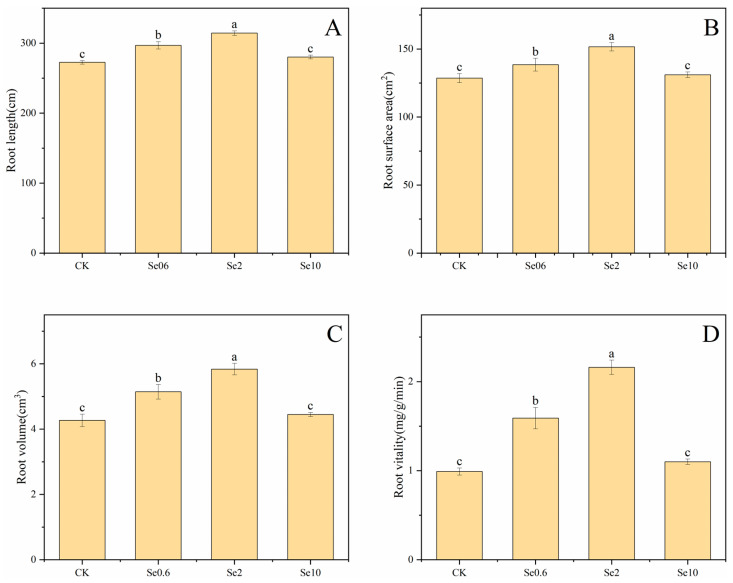
Effect of applied selenium on root length (**A**), root surface area (**B**), root volume (**C**), and root activity (**D**) of prickly pear. Note: CK-0 mg/kg soil Se application; Se0.6-0.6 mg/kg soil Se application. Se2-2 mg/kg soil Se application. Se10-10 mg/kg soil Se application. Different lowercase letters indicate significant differences between treatments and CK at 0.05 level.

Soil Se affected the morphological characteristics and root activity of prickly pear (Figure 1). The root length, root surface area, root volume, and root activity increased significantly in the Se0.6 and Se2 treatments compared to CK (*p* < 0.05). In the Se2 treatment, the root length, root surface area, and root volume of prickly pear were 15%, 17%, and 37% higher than those in CK, respectively. The root activity of prickly pear significantly increased by 64% and 128% in the Se0.6 and Se2 treatments, respectively, compared to CK (*p <* 0.05). There were no significant differences in root length, root surface area, root volume, or root activity between the Se10 treatment and CK.

### 4.2. Effects of Se on the Photosynthetic Pigments in Prickly Pear Leaves

**Figure 2:**
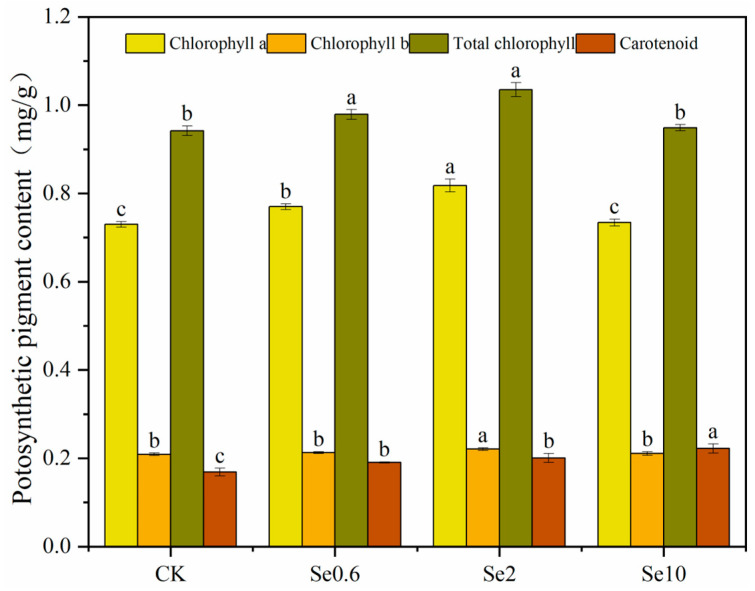
Effect of applied selenium on photosynthetic pigment of prickly pear. Note: CK-0 mg/kg soil Se application; Se0.6-0.6 mg/kg soil Se application. Se2-2 mg/kg soil Se application. Se10-10 mg/kg soil Se application. Different lowercase letters indicate significant differences between treatments and CK at 0.05 level.

The photosynthetic pigments in prickly pear leaves showed significant differential responses to soil Se (Figure 2). The chlorophyll a, chlorophyll b, and total chlorophyll in prickly pear leaves first increased and then decreased with increasing Se content in the soil. The leaves’ chlorophyll a, chlorophyll b, and total chlorophyll contents were maximal in the Se2 treatment and showed significant increases of 12%, 6%, and 10%, respectively, compared to CK (*p* < 0.05). The total chlorophyll content in the Se10 treatment showed no significant difference compared to CK. The carotenoid contents in the Se0.6, Se2, and Se10 treatments significantly increased by 14%, 23%, and 32% (*p* < 0.05), respectively, compared to CK.

### 4.3. Effects of Se on Enzymes Activity, MDA, and Soluble Protein in Prickly Pear Leaves

**Figure 3:**
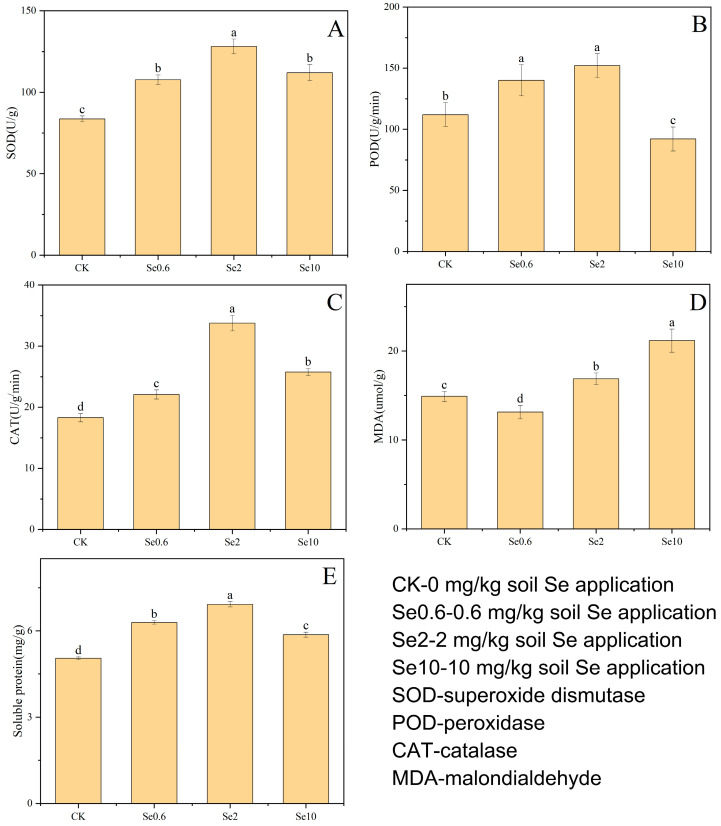
Effect of Se application on SOD (**A**), POD (**B**), CAT (**C**), MDA (**D**) and soluble protein (**E**) of prickly pear leaves. Different lowercase letters indicate significant differences between treatments and CK at 0.05 level.

The soil Se treatments increased the SOD, POD, and CAT activity in prickly pear leaves, except for the Se10 treatment (Figure 3). The maximum SOD activity of 128.13 U/g was recorded in the Se2 treatment (*p* < 0.05). The POD activity in prickly pear leaves was increased by 25% and 36% in the Se0.6 and Se2 treatments, respectively, compared to CK, whereas it was lower than the CK value in the Se10 treatment (*p* < 0.05). The CAT activity in Se0.6, Se2, and Se10 was increased by 21%, 85%, and 38%, respectively, compared to CK. The MDA contents in the Se2 and Se10 treatments were significantly increased compared to that in CK. However, the MDA content in the Se0.6 treatment was decreased compared to that in CK, indicating that low Se levels reduced the malondialdehyde accumulation (*p* < 0.05). The soluble protein contents of prickly pear leaves in the three treatments increased significantly compared to that in CK (*p* < 0.05), with a trend of first increasing and then decreasing with the increase in soil Se content.

### 4.4. Effect of Se on Se Allocation in Prickly Pear

**Figure 4:**
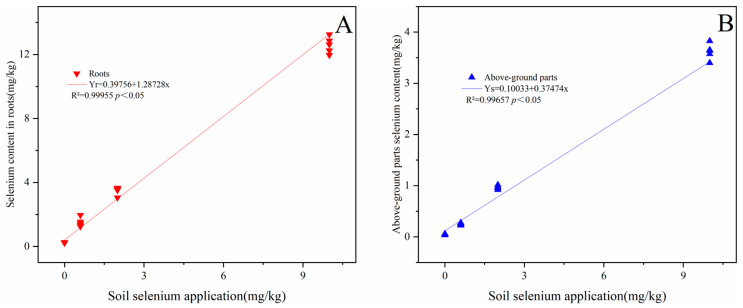
Relationship between Se content in roots vs. soil Se application (**A**) and aboveground Se vs. soil Se application (**B**). Note: Yr—Se content in roots; Ys—Se content in aboveground parts; x—soil Se application.

With the increase in soil selenium content, the selenium contents of prickly pear roots, stems, and leaves were significantly increased compared to CK (*p* < 0.05) (Table 2). The Se contents in prickly pear roots were significantly linearly correlated with the soil’s Se content (R^2^ = 0.9996, *p* < 0.05) (Figure 4). The Se contents in the aboveground parts were also significantly linearly correlated with the soil’s Se content (R^2^ = 0.9966, *p* < 0.05). The translocation factor of prickly pear for Se was less than one when the soil’s Se content ranged from 0 to 10 mg/kg. The translocation factor of prickly pear in the Se2 and Se10 treatments was significantly higher than that in CK and the Se0.6 treatment (*p* < 0.05), indicating that the soil Se content increased the translocation factor of prickly pear for Se.

### 4.5. Effects of Se on the Rhizosphere Soil Properties of Prickly Pear

**Figure 5:**
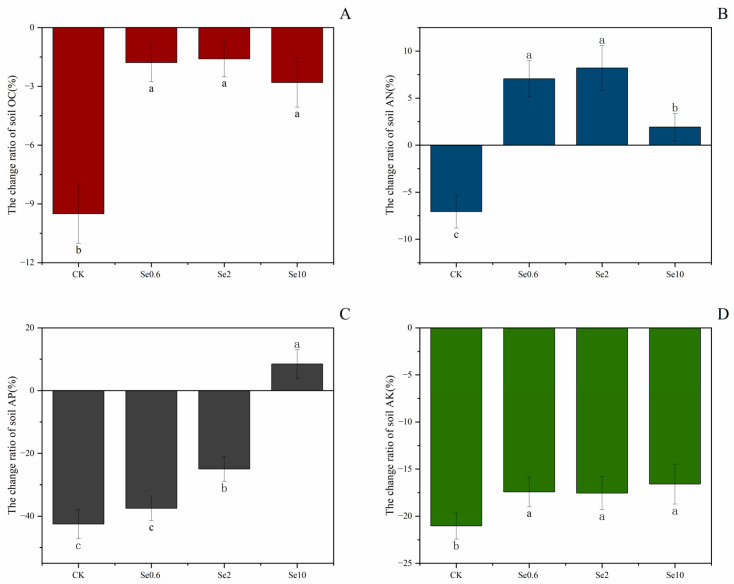
Effect of Se application on change rate of soil OC (**A, deep maroon**), AN (**B, dark blue**), AP (**C, black**) and AK (**D, Green**). Note: CK-0 mg/kg of soil Se application. Se0.6-0.6 mg/kg soil Se application. Se2-2 mg/kg soil Se application. Se10-10 mg/kg soil Se application. OC-organic carbon. AN-alkali-dissolved N. AP-available P. AK-available K. Different lowercase letters indicate significant differences between treatments and CK at 0.05 level.

The soil organic C and nutrient contents in the Se treatments and CK changed in different patterns after the pot experiment (Figure 5). The change ratios of soil organic C under the Se0.6, Se2, and Se10 treatments and CK were negative after the pot experiment. The Se treatments showed smaller reductions in soil organic C compared with CK (*p* < 0.05). The change ratios of the soil’s alkali-dissolved N contents in the Se0.6, Se2, and Se10 treatments were positive, while they were negative for CK. The Se treatments significantly increased the soil’s alkali-dissolved N content compared with CK (*p* < 0.05). Only the Se10 treatment showed a positive change ratio for the soil’s available P. The Se2 treatment significantly slowed down the decrease in the soil’s available P compared with CK and Se0.6 (*p* < 0.05). The change ratios for the soil’s available K in the Se treatments and CK were negative. Compared with CK, the soil’s available K decreased less with the application of Se to the soil (*p* < 0.05). The Se2 and Se10 treatments significantly increased the soil’s pH, by 4% and 7% (*p* < 0.05), respectively, compared to CK (Supplementary Figure S2). There were no significant differences in rhizosphere soil conductivity between the three treatments and CK.

### 4.6. Effect of Se on the Methane Oxidation in Rhizosphere Soil

**Figure 6:**
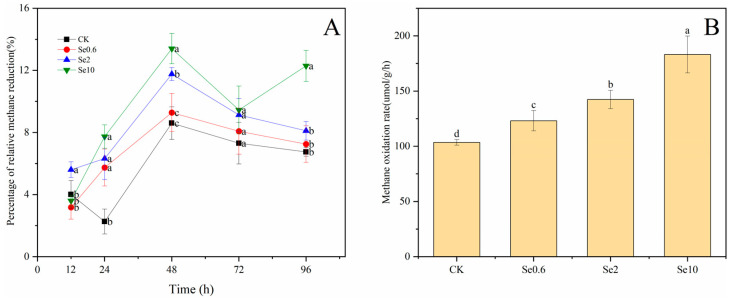
Relative methane reduction (**A**) and methane oxidation rate (**B**) of rhizosphere soil of prickly pear. Note: CK-0 mg/kg soil Se application. Se0.6-0.6 mg/kg soil Se application. Se2-2 mg/kg soil Se application. Se10-10 mg/kg soil Se application. Different lowercase letters indicate significant differences between different treatments and the control at 0.05 level. Different letters in the same column indicate significant differences between treatments and CK at 0.05 level.

Soil selenium enhanced the methane oxidation in prickly pear rhizosphere soil (Figure 6). At 12 h, the relative methane reduction in the Se2 treatment was significantly higher than that in CK (*p* < 0.05), whereas the relative methane reduction in the Se0.6 and Se10 treatments was not significantly changed. At 24 h, the relative methane reduction in the three treatments was significantly higher than that in CK (*p* < 0.05). At 48 h, the relative methane reduction in the Se0.6, Se2, and Se10 treatments reached maximum values of 12%, 12%, and 14%, respectively. There were no significant differences in the methane reduction between the treatments and CK at 72 h. The relative methane reduction in the Se10 treatment showed a maximum value at 96 h (*p* < 0.05). Meanwhile, significant differences in methane oxidation rates were observed between the three treatments and CK after 96 h of incubation (*p* < 0.05). The methane oxidation rates of the rhizosphere soils increased with the increase in Se content. The Se10 treatment had the maximum oxidation rate of 183 nmol/g/h.

### 4.7. Overall Effects of Se on Growth, Soil Properties, and Methane Oxidation

**Figure 7:**
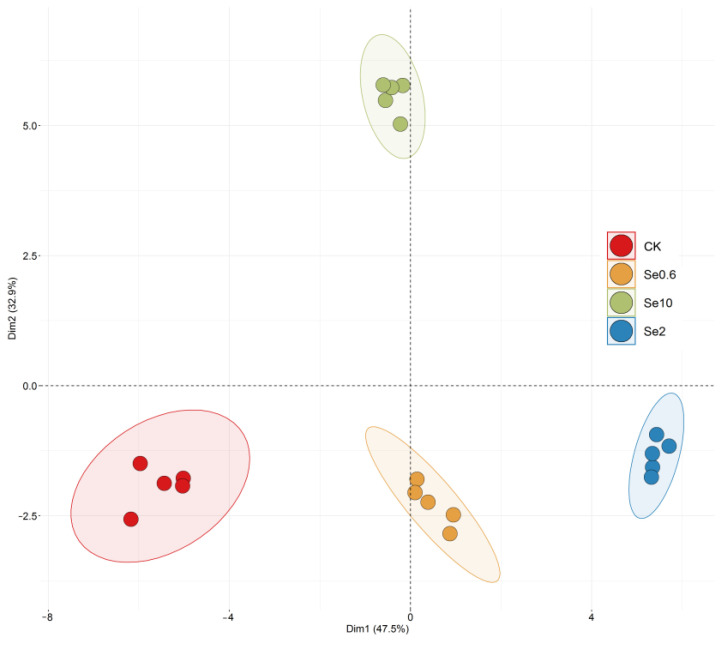
Principal component analysis of soil selenium effects on prickly pear. Note: CK-0 mg/kg soil Se application. Se0.6-0.6 mg/kg soil Se application. Se2-2 mg/kg soil Se application. Se10-10 mg/kg soil Se application.

The overall effects of Se were analyzed through PCA for Se0.6, Se2, Se10, and CK (Figure 7). The variance contributions of the extracted PCA1 and PCA2 were 47.5% and 32.9%, respectively, and the cumulative variance contribution was 80%. The contribution values of Se0.6 and Se2 to PCA1 were positive. Se10 had a positive value and the largest contribution to PCA2. 

**Figure 8:**
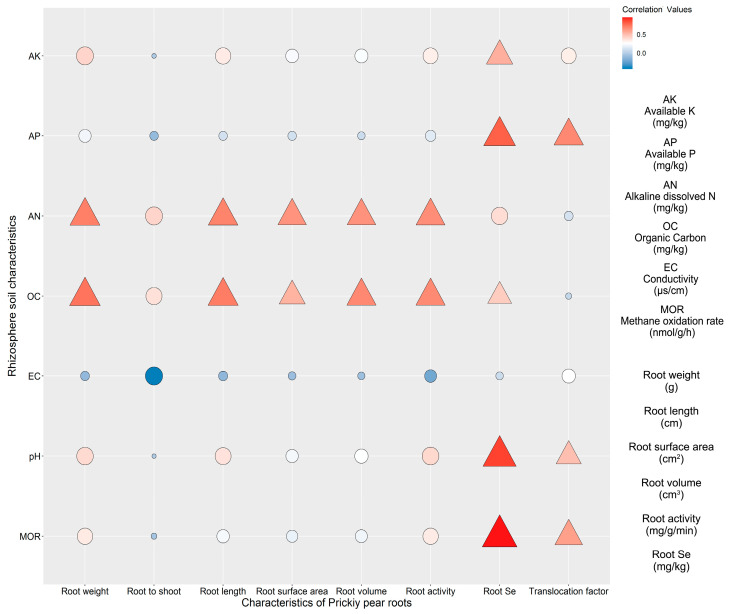
Correlation analysis between soil properties and roots characteristics of prickly pear. Note: Triangle represents significant value and Circle represents insignificant value at 0.05 level.

Spearman’s correlation analysis was used to study the relationships between the prickly pear root characteristics and final soil properties (Figure 8). The correlation between various indicators of the prickly pear root system and the final soil organic C and final alkali-dissolved N was strong, in which the root weight, root length, root volume, and root surface area were all significantly positively correlated with the final soil organic C and final alkali-dissolved N (*p* < 0.05). The root activity was also significantly positively correlated with the final soil organic C and final alkali-dissolved N content, while the final soil pH, final soil organic C, final soil available P, and final available K showed a significant positive correlation with the roots’ Se content (*p* < 0.05). The Se translocation factor was also positively correlated with the final soil pH and final available P. In addition, the roots’ Se content and the Se translocation factor were significantly positively correlated with the methane oxidation rate (*p* < 0.05).

## 5. Discussion

### 5.1. Effect of Se on Prickly Pear Growth

Se promotes plant growth at low concentrations, but excessive Se is toxic to plants [30]. In this study, soil Se induced changes in the growth characteristics of prickly pear and the properties of its rhizosphere soil. Low application of Se to soil (0.6 mg/kg, 2 mg/kg) significantly increased the biomass of the roots and aboveground parts of the prickly pear. Furthermore, the PCA results indicated that significant differentiation occurred between the different treatments in terms of soil Se content. The direct interaction between the Se and prickly pear took place in the roots, and low Se contents significantly increased the root activity, which may have been related to the promotion of root growth by Se [51]. As Se constructs proteins in the form of selenoamino acids, which nonspecifically replace sulfur, excessive Se leads to protein inactivation [24]. As the Se content was increased to 10 mg/kg, the promotional effect of Se on the prickly pear roots weakened. There were no significant differences in the biomass of the roots compared to CK, and no toxic effects on the prickly pear plants were observed. Studies have found that when the concentration of sodium selenite is 0.5 mg/kg or 1 mg/kg, the root activity of alfalfa is significantly enhanced, but when it reaches 5 mg/kg or more, the root activity is inhibited [30]. We found that prickly pear was able to tolerate a certain level of Se in its soil. The roots of prickly pear may convert Se to selenomethylcysteine and other non-protein amino acids, reducing the toxicity of high Se concentrations [52].

### 5.2. Effects of Se on the Photosynthetic Pigments in Prickly Pear Leaves

Photosynthetic pigment content is an essential factor indirectly reflecting plant growth [53]. We found that 0.6 mg/kg and 2 mg/kg applications of Se to the soil increased the photosynthetic pigment contents in prickly pear leaves. Se enhancement effects have also been reported in garlic, barley, and alfalfa [30,54,55]. Se can regulate mineral element absorption, enzyme activity, and the respiration rate, contributing to increased chlorophyll biosynthesis. In a previous study, the addition of Se increased the chlorophyll a and b contents in *Lycium chinense* leaves by stimulating the respiration rate and the flow of electrons in the respiratory chain [29]. Low Se concentration contributed to an increase in the contents of chlorophyll a, chlorophyll b, and total chlorophylls of spinach [56]. Excessive Se decreased the photosynthetic efficiency [57], possibly due to damage to the structure of photosystem II complexes in chloroplasts, chlorophyll degradation, and oxidative stress.

The higher the relative chlorophyll content, the more light energy is converted by photosynthesis [53]. We found that the chlorophyll a/b ratio of prickly pear increased in the 0.6 and 2 mg/kg treatments, indicating that low Se contents in soil are beneficial for the photosynthesis of prickly pear leaves. Carotenoids protect plants from photo-oxidative damage and maintain membrane structure stability [58]. In this study, the contents of carotenoids in leaves increased with the increase in soil Se content. The reduction in carotenoid concentration induced by high Se contents in soil could lead to chlorophyll oxidation, negatively affecting photosynthetic efficiency [59]. The changes in carotenoid content were similar to the changes in malondialdehyde content in prickly pear leaves in each treatment, possibly because carotenoids help eliminate peroxyl radicals and singlet molecular oxygen in prickly pear leaves [30,60].

### 5.3. Effects of Se on Enzyme Activity, MDA, and Soluble Protein in Prickly Pear Leaves

Se regulates the levels of reactive oxygen species in plants by regulating antioxidant enzymes such as SOD, POD, and CAT [61]. Low concentrations of Se increase the abundance of antioxidant enzymes, while high concentrations lead to oxidative stress [62]. The appropriate Se application (2 mg/kg) significantly enhanced the activities of POD, CAT, and SOD in prickly pear. The economically important walnut (*Juglans regia* L.) showed a significant increase in SOD enzyme activity when treated with a foliar spray containing 30 mg/L Se [63]. The increase in POD levels is related to the excess H_2_O_2_ generated by the increase in SOD levels [30]. The degree of peroxidation increases with the MDA content [64]. We found that the MDA content in prickly pear leaves was significantly reduced under the 0.60 mg/kg Se treatment compared to CK. Low Se contents enhanced the antioxidant enzyme activity, clearing MDA produced by membrane lipid peroxidation, which helps protect the membrane integrity of cells [65]. As the soil’s Se content increases, excessive accumulation of MDA damages the integrity and function of cell membranes.

Prickly pear leaves are rich in free amino acids, making them an essential raw material for Chinese tea [17]. This study has demonstrated that soil Se increased the soluble protein content in leaves, indicating that appropriate levels of Se can improve the leaf quality of prickly pear. Furthermore, previous research demonstrated that supplementation with sodium selenite, whether through soil addition, trunk injection, or leaf spraying, resulted in an increase in the soluble protein content of jujube fruits [66]. Furthermore, inorganic Se is generally transformed into amino acids such as selenocysteine (S-Cys) and selenomethylmethionine (SE-Met) [61,67], which are directly involved in plant protein synthesis. Excessive Se impairs plants’ protein formation under Se stress [68]. In this study, the soluble protein content in prickly pear leaves increased with increasing Se content in the soil, and Se showed the potential to improve the nutritional quality of prickly pear leaves.

### 5.4. Effect of Se on Se Accumulation in Prickly Pear

The content and speciation of Se in soil affect the roots’ Se uptake efficiency. We found that there was a significant linear positive correlation between the Se content in both the root and aboveground parts of prickly pear and the soil Se content, indicating that prickly pear has the potential to enrich Se in soil. Selenite can penetrate root cells through passive diffusion [69], accounting for the occurrence of Se accumulation in roots in soil with high selenite stress. Other studies [68,70] have found that selenite uptake by the plant root system is an active process, which may be partially mediated by phosphate transport proteins. In addition, prickly pear roots may take up selenate and organic Se compounds (e.g., SeCys2, SeMet, and MeSeCys) that have been transformed from selenite in the soil through microbial metabolism [24]. Subsequently, these organic Se compounds in roots are transported to the aboveground parts of the prickly pear [71]. The Se uptake rate in prickly pear roots may be greater than the conversion rate, resulting in a Se transport coefficient of less than one [71]. When lettuce (*Lactuca sativa*) is supplied with selenite, it is taken up by the roots and transformed into selenate and organic Se compounds, which are then transported to the aboveground parts [72].

### 5.5. Effects of Se on the Rhizosphere Soil Properties of Prickly Pear

Rhizosphere soil indicators directly affect the physiological metabolism of roots [73]. In this study, there was a correlation between the physicochemical properties of rhizosphere soil and the growth characteristics of prickly pear roots. Soil Se may affect the microhabitat of prickly pear roots through the increased pH and change soil organic C, alkali-dissolved N, available P, and available K in the rhizosphere soil. We found that the final organic C content in the rhizosphere soil of prickly pear was always lower than that at the beginning. However, the final organic C content in the Se treatments was higher than that in CK, indicating a slow reduction in organic C after the application of Se. A previous study showed that Se increased the soluble organic C content of rapeseed rhizosphere soil [74]. Se can also enhance the formation of root exudates by improving plant photosynthesis and releasing more soluble organic C into the rhizosphere soil [75,76]. In addition, Se is involved in the metabolism of methane-oxidizing bacteria [77]. In this study, the methane oxidation potential of soil treated with Se was significantly enhanced. During the microbial methane oxidation process, about 23.6–60% of the consumed methane was converted to soil organic C, and methanotroph-mediated synthesis of biomass plays an important role in the accumulation of soil organic matter [78]. This phenomenon indicates that Se promotes the accumulation of organic C while promoting methane oxidation in the rhizosphere soil, which partially explains the slowdown in the reduction in organic C in the Se treatments.

The changes in functional microbial communities such as N-fixation microbes and K-solubilization microbes, help increase the contents of alkali-dissolved N and available potassium in rhizosphere soil. P and Se share transporter proteins, and the P uptake efficiency of prickly pear may be affected by the addition of Se. The absorption of selenite by wheat increased by 60% in a low-P environment [71]. Sodium selenite saturates the transporter and competes with P for the site [30]. Therefore, with the increase in soil selenite content, the soil’s available P content decreased less, which led to higher soil-available P contents in the Se treatments compared with CK. Se reduces the secretion of organic acids such as oxalic, malic, malonic, tartaric, and acetic acids in plant roots, leading to an increase in the rhizosphere soil’s pH [79]. We speculated that Se had affected the secretion of organic acids from the prickly pear roots. It is worth noting that increasing the pH in rhizosphere soil helps to enhance the efficiency of soil Se uptake by plant roots [80], and the pH increase in the rhizosphere soil may, in turn, improve the Se uptake of prickly pear.

### 5.6. Effect of Se on the Methane Oxidation in Rhizosphere Soil

In this study, Se significantly promoted methane oxidation in rhizosphere soils, in the following order: Se10 > Se2 > Se0.6 > CK. A variety of electron acceptors can participate in methane metabolism processes mediated by methane-oxidizing bacteria. Methane-oxidizing bacteria can couple anaerobic methane oxidation with the reduction process of NO_2_^−^, NO_3_^−^, SO_4_^2−^, and SeO_4_^2−^, which are also electron acceptors for methane-oxidizing bacteria [81,82,83]. With methane as the only electron donor, *Methylomonas* sp. directly oxidizes methane and reduces SeO_4_^2−^ [27,77]. The SeO_3_^2-^ may be converted to Se in rhizosphere soil and participate in the methane oxidation process as an electron acceptor. The methanotroph model strains *Methylococcus capsulatus* (Bath) and *Bacillus trichosporus* OB3b can reduce SeO_3_^2−^ to red spherical nano-Se [26]. In this study, there was a significant positive relationship between Se and rhizosphere nutrient contents. The increase in nutrient contents in the rhizosphere greatly enhanced the activity of the microbial community, and methanotrophs were beneficial for the environmental changes in the rhizosphere soil of prickly pear. As a result, the methane oxidation process in the rhizosphere soil of prickly pear was influenced as follows. However, there is currently a lack of research on the relationship between methanotrophs and Se’s speciation and transformation in the rhizosphere soil of prickly pear. Multiple omics methods combined with root metabolism and microbial community diversity can be used to further elucidate the effects of Se on the rhizosphere physiological ecology and methane oxidation mechanism of prickly pear.

### 5.7. Overall Effects of Se on Growth, Soil Properties, and Methane Oxidation

In this study, exogenous application of Se showed a differentiated overall effect on the soil–prickly-pear system, based on the PCA results. The application of Se altered the growth of prickly pear. A previous study found that the application of Se (≥1 mg/kg) significantly improved the growth of polysavone and produced toxic effects when the amount of Se exceeded the threshold (20 mg/kg) [30]. In addition, proper amounts of exogenous Se increased the root biomass, root morphological traits, and root activity of prickly pear. We found that Se interacted strongly with the rhizosphere soil habitat of prickly pear. Due to the overall responses of the rhizosphere soil properties, Se caused changes in soil methane oxidation. After Se was applied to the soil, the Se translocation factor showed a close relationship with the soil properties and methane oxidation. The application of Se can alter the functional microbial groups in soil [74], which are always involved in the soil properties’ responses and the methane metabolism process [84,85]. Furthermore, changes in the physical and chemical properties of the soil can also affect the level of soil methane metabolism [86].

Se plays a positive role in promoting the growth, nutritional quality, and low-carbon effect of prickly pear. Transcriptomics tools can be used to analyze the functional genes and metabolic responses of prickly pear under Se supplementation. In addition, the effects of different species of Se on bioactive ingredients such as vitamins and polysaccharides in prickly pear are essential, which can provide more theoretical support for the Se-rich prickly pear industry.

## 6. Conclusions

Prickly pear is a cash crop unique to Asia, especially in underdeveloped areas. In this study, we studied the effects of soil Se on the growth and methane oxidation of prickly pear, suggesting that the cultivation of prickly pear in Se-rich soils has economic and ecological benefits. Our conclusions are as follows: (1) Low soil Se has a strong positive effect on the growth and nutritional quality of prickly pear leaves, whereas the promotional effect is weakened under high soil Se contents. (2) Increased soil Se improves the Se translocation in prickly pear, proving that prickly pear can accumulate Se in Se-rich soil. (3) Se can improve the nutrient status and methane oxidation capacity of the rhizosphere soil of prickly pear. This study revealed the physiological responses of prickly pear to Se and the low-carbon effect of Se-enriched prickly pear production. The large-scale cultivation of prickly pear in Se-enriched soil has economic and potential ecological benefits.

## Figures and Tables

**Table 1:** Effect of selenium application on biomass of prickly pear.

Group	Total Biomass (g/Plant)	Root Biomass (g/Plant)	Stem Biomass (g/Plant)	Leaf Biomass (g/Plant)	Root/Shoot
CK	9.029 ± 0.115 d	3.355 ± 0.021 c	4.440 ± 0.085 b	1.234 ± 0.013 c	0.591 ± 0.007 bc
Se0.6	9.478 ± 0.074 b	3.522 ± 0.050 b	4.596 ± 0.0230 a	1.360 ± 0.070 b	0.591 ± 0.006 b
Se2	9.852 ± 0.076 a	3.862 ± 0.045 a	4.584 ± 0.060 a	1.406 ± 0.009 a	0.646 ± 0.009 a
Se10	9.212 ± 0.039 c	3.406 ± 0.011 c	4.556 ± 0.045 a	1.254 ± 0.011 b	0.586 ± 0.004 c

Note: CK-0 mg/kg soil Se application; Se0.6-0.6 mg/kg soil Se application. Se2-2 mg/kg soil Se application. Se10-10 mg/kg soil Se application. Different lowercase letters in the same column indicate significant differences between treatments and CK at 0.05 level.

**Table 2:** Selenium content and translocation factor of prickly pear under soil selenium contents.

Group	Root (mg/kg)	Stem (mg/kg)	Leaf (mg/kg)	Aboveground (mg/kg)	Translocation Factor
CK	0.245 ± 0.015 d	0.038 ± 0.010 c	0.088 ± 0.004 c	0.048 ± 0.008 d	0.199 ± 0.041 b
Se0.6	1.504 ± 0.247 c	0.230 ± 0.024 c	0.334 ± 0.014 c	0.254 ± 0.019 c	0.173 ± 0.029 b
Se2	3.488 ± 0.224 b	0.600 ± 0.034 b	2.160 ± 0.224 b	0.966 ± 0.031 b	0.278 ± 0.012 a
Se10	12.588 ± 0.452 a	2.466 ± 0.175 a	7.798 ± 1.142 a	3.617 ± 0.138 a	0.288 ± 0.019 a

Note: CK-0 mg/kg soil Se application; Se0.6-0.6 mg/kg soil Se application. Se2-2 mg/kg soil Se application. Se10-10 mg/kg soil Se application. Different lowercase letters indicate significant differences between treatments and CK at 0.05 level.

## Data Availability

https://figshare.com/s/e0f9ab5986052434df65.

## Supplementary Materials

The following supporting information can be downloaded at: https://www.mdpi.com/article/10.3390/plants13060749/s1. Supplementary tables and figures were supplied as a separate file. Table S1: Basic soil properties; Figure S1: Effect of soil selenium on height (A) and stem diameter (B) of prickly pear; Figure S2: Effect of different selenium on soil pH (A) and conductivity (B).

## References

[1] Chen N., Zhao C.H., Zhang T.H. (2021). Selenium transformation and selenium-rich foods. Food Biosci..

[2] Wadgaonkar S.L., Nancharaiah Y.V., Esposito G., Lens P.N.L. (2018). Environmental impact and bioremediation of seleniferous soils and sediments. Crit. Rev. Biotechnol..

[3] Zhou X.B., Yang J., Kronzucker H.J., Shi W.M. (2020). Selenium Biofortification and Interaction with Other Elements in Plants: A Review. Front. Plant Sci..

[4] Wang Z.Y., Ding Y.Z., Ren X.A., Xie J.W., Kumar S., Zhang Z.Q., Wang Q. (2022). Effect of micronutrient selenium on greenhouse gas emissions and related functional genes during goat manure composting. Bioresour. Technol..

[5] Duborská E., Sebesta M., Matulová M., Zverina O., Urík M. (2022). Current Strategies for Selenium and Iodine Biofortification in Crop Plants. Nutrients.

[6] Yang X.F., Ning Z.P., Kwon S.Y., Li M.L., Tack F.M.G., Kwon E.E., Rinklebe J., Yin R.S. (2022). The beneficial and hazardous effects of selenium on the health of the soil-plant-human system: An overview. J. Hazard. Mater..

[7] Sun M.F., Wang J.J., Liu W., Yin P., Guo G.Y., Tong C.L., Chang Y.L. (2022). Effect and mechanism of exogenous selenium on selenium content and quality of fresh tea leaves. Not. Bot. Horti Agrobot..

[8] Tang W., Tang W.J., Xie Y.D., Li X.M., Li H.X., Lin L.J., Huang Z., Sun B., Sun G.C., Tu L.H. (2023). Effects of intercropping on Se accumulation and growth of pakchoi, lettuce and radish. Int. J. Phytoremediat..

[9] Yao F.X., Wen L., Chen R., Du C., Su S.M., Yan M.M., Yang Z.L. (2022). Enrichment characteristics and dietary evaluation of selenium in navel orange fruit from the largest navel orange-producing area in China (southern Jiangxi). Front. Plant Sci..

[10] Ning P., Fei P.W., Wu T.Q., Li Y.F., Qu C.Y., Li Y.N., Shi J.L., Tian X.H. (2022). Combined foliar application of zinc sulphate and selenite affects the magnitude of selenium biofortification in wheat (*Triticum aestivum* L.). Food Energy Secur..

[11] Liu J., Mo A., Ni J., Fan X., Jiang Y. (2023). Selenium Reduces Rice Plant Tissues Cadmium and Increases the Yield, Quality, and Edible Safety of Rice Grain, and May Affect the Taste of Cooked Rice. J. Soil Sci. Plant Nutr..

[12] Zhi H., Xia Q., Yang Z.P., Gao Z.Q. (2023). Effects of selenium through soil application on soil fertility and grain quality in winter wheat. Appl. Ecol. Environ. Res..

[13] de los Angeles Sarinana-Navarrete M., Guillermo Hernandez-Montiel L., Sanchez-Chavez E., Jose Reyes-Perez J., Murillo-Amador B., Reyes-Gonzalez A., Preciado-Rangel P. (2021). Foliar fertilization of sodium selenite and its effects on yield and nutraceutical quality in grapevine. Rev. Fac. Agron..

[14] Xu J.W., Vidyarthi S.K., Bai W.B., Pan Z.L. (2019). Nutritional constituents, health benefits and processing of Roxburghii: A review. J. Funct. Foods.

[15] Huang C.Y., Cui L.Y., Lin F.Q. (2023). Chemical Constituents and Research Progress on Biological Functions of *Rosa roxburghii*. Food Ind..

[16] Luo Z.H., Wang B., Huang Q., Li Y.Y., Guo P.P., Zeng G.L., Zhang C.L., Song C. (2020). Geochemical Characterristics of Soil Selenium and Its Application in Selenium-rich Agriculture in Shuicheng County, Guizhou Province. Guizhou Deology.

[17] Song Q., Niu S., Yang Q., Fan J., Yin J. (2022). Study and Evaluation on Quality of Rosa roxburghii Leaves Tea with Different Processing Technology. Farm. Prod. Process..

[18] Zhang C., Yan K., Lin L.Z., Fang Y.M., Zhang X.Y. (2022). Effects of source-sink alteration by pruning on physiological parameters and fruit production of Tratt. on the Yunnan-Guizhou Plateau in China. Photosynthetica.

[19] Shakoor A., Ashraf F., Shakoor S., Mustafa A., Rehman A., Altaf M.M. (2020). Biogeochemical transformation of greenhouse gas emissions from terrestrial to atmospheric environment and potential feedback to climate forcing. Environ. Sci. Pollut. R..

[20] Knief C. (2019). Diversity of Methane-cycling Microorganisms in Soils and Their Relation to Oxygen. Curr. Issues Mol. Biol..

[21] Cui J., Zhang M., Chen L.X., Zhang S.H., Luo Y., Cao W.W., Zhao J., Wang L.X., Jia Z.J., Bao Z.H. (2022). Methanotrophs Contribute to Nitrogen Fixation in Emergent Macrophytes. Front. Microbiol..

[22] Yang K., Ji H., Xing Z.X., Liu G.C., Yu S.W., Gu J.X. (2016). Influence of Methanogens and Methanotrophs on Methane Generation and Oxidation in the Plant-Soil Ecological System. Oxid. Commun..

[23] Gar’kusha D.N., Fedorov Y.A. (2016). Effect of plants on processes of methane cycle in bottom deposits and soil rhizosphere. Contemp. Probl. Ecol..

[24] Schiavon M., Pilon-Smits E.A.H. (2017). The fascinating facets of plant selenium accumulation—Biochemistry, physiology, evolution and ecology. New Phytol..

[25] Feng R.W., Wang L.Z., Yang J.G., Zhao P.P., Zhu Y.M., Li Y.P., Yu Y.S., Liu H., Rensing C., Wu Z.Y. (2021). Underlying mechanisms responsible for restriction of uptake and translocation of heavy metals (metalloids) by selenium via root application in plants. J. Hazard. Mater..

[26] Eswayah A.S., Smith T.J., Scheinost A.C., Hondow N., Gardiner P.H.E. (2017). Microbial transformations of selenite by methane-oxidizing bacteria. Appl. Microbiol. Biot..

[27] Luo J.H., Chen H., Hu S.H., Cai C., Yuan Z.G., Guo J.H. (2018). Microbial Selenate Reduction Driven by a Denitrifying Anaerobic Methane Oxidation Biofilm. Environ. Sci. Technol..

[28] Shi L.D., Lv P.L., McIlroy S.J., Wang Z., Dong X.L., Kouris A., Lai C.Y., Tyson G.W., Strous M., Zhao H.P. (2021). Methane-dependent selenate reduction by a bacterial consortium. ISME J..

[29] Dong J.Z., Wang Y., Wang S.H., Yin L.P., Xu G.J., Zheng C., Lei C., Zhang M.Z. (2013). Selenium increases chlorogenic acid, chlorophyll and carotenoids of Lycium chinense leaves. J. Sci. Food Agric..

[30] Bai B., Wang Z., Gao L., Chen W., Shen Y. (2019). Effects of senlenite on the growth of alfalfa (*Medicogo sativa* L. cv. Sadie 7) and related physiological mechanisms. Acta Physiol. Plant.

[31] Zhang Z.M., He H.Z., Zhang J.C., Li Q., Liu Y.Y. (2016). Effects of Ectomycorrhiza on the growth and distribution of carbon, nitrogen and phosphorus in seedless prickly pear. North Hortic..

[32] Dai H.P., Jia G.L. (2017). Effects of Se on the growth, tolerance, and antioxidative systems of three alfalfa cultivars. Environ. Sci. Pollut. R..

[33] Lu F., Bu Z.J., Lu S. (2019). Estimating Chlorophyll Content of Leafy Green Vegetables from Adaxial and Abaxial Reflectance. Sensors.

[34] Aebi H.J.M. (1984). Catalase in vitro. Methods Enzymol..

[35] Zhou Z.S., Wang S.J., Yang Z.M. (2008). Biological detection and analysis of mercury toxicity to alfalfa (*Medicago sativa*) plants. Chemosphere.

[36] Jiang L., Ma L., Sui Y., Han S.Q., Wu Z.Y., Feng Y.X., Yang H. (2010). Effect of manure compost on the herbicide prometryne bioavailability to wheat plants. J. Hazard. Mater..

[37] Feng R.W., Wei C.Y. (2012). Antioxidative mechanisms on selenium accumulation in *Pteris vittata* L. A potential selenium phytoremediation plant. Plant Soil Environ..

[38] Rekowski A., Langenkämper G., Dier M., Wimmer M.A., Scherf K.A., Zörb C. (2021). Determination of soluble wheat protein fractions using the Bradford assay. Cereal Chem..

[39] Tavares O.C.H., Santos L.A., Ferreira D., Ferreira L.M., García A.C., Castro T.A.V., Zonta E., Pereira M.G., Fernandes M.S. (2021). Response surface modeling of humic acid stimulation of the rice (*Oryza sativa* L.) root system. Arch. Agron. Soil Sci..

[40] Wang C.Z., Zhang D.J., Zhang J., Ji T.W., Tang Z.Q., Zhao Y.Y. (2015). Allelopathic effects of volatile compounds from Eucalyptus grandis on *Vigna radiata*, *Raphanus sativus* and *Lactuca sativa*. Allelopathy J..

[41] Bao S.D. (2000). The Agricultural Chemical Analysis Method of Soil.

[42] Lu R.K. (2000). Analytical Methods of Soil Agrochemistry.

[43] Jones J.B. (1973). Soil testing in the united states. Commun. Soil Sci. Plan..

[44] Ni R.X., Luo K.L., Tian X.L., Yan S.G., Zhong J.T., Liu M.Q. (2016). Distribution and geological sources of selenium in environmental materials in Taoyuan County, Hunan Province, China. Environ. Geochem. Health.

[45] Zhang Z.H., Rengel Z., Meney K. (2010). Cadmium Accumulation and Translocation in Four Emergent Wetland Species. Water Air Soil Poll..

[46] Wang Y.F., Cai Y.F., Hou F.J., Saman B., Jia Z.J. (2023). Grazing Effect on Activity and Diversity of Soil Methanotrophs in Winter Pastures of the Loess Plateau. Acta Pedol. Sin..

[47] Gultekin R., Avag K., Görgisen C., Öztürk Ö., Yeter T., Bahçeci Alsan P. (2023). Effect of deficit irrigation practices on greenhouse gas emissions in drip irrigation. Sci. Hortic..

[48] Gilleland E., Viii G., R Development Core Team (2014). R: A Language and Environment for Statistical Computing.

[49] Kassambara A., Mundt F. (2017). Factoextra: Extract and Visualize the Results of Multivariate Data Analyses.

[50] Le S., Josse J., Husson F. (2008). FactoMineR: An R package for multivariate analysis. J. Stat. Softw..

[51] Li Y., Xiao Y.R., Hao J.H., Fan S.X., Dong R.F., Zeng H.H., Liu C.J., Han Y.Y. (2022). Effects of selenate and selenite on selenium accumulation and speciation in lettuce. Plant Physiol. Biochem..

[52] Yuan L.X., Zhu Y.Y., Lin Z.Q., Banuelos G., Li W., Yin X.B. (2013). A Novel Selenocystine-Accumulating Plant in Selenium-Mine Drainage Area in Enshi, China. PLoS ONE.

[53] Xu L.P., Zhang L.Y., Yi B., Zhang Z.Q. (2022). Genetic dissection of photosynthetic pigment content diversity and identification of loci associated with photoperiod and alkaline soil responses. Ind. Crop Prod..

[54] Dumont E., Vanhaecke F., Cornelis R. (2006). Selenium speciation from food source to metabolites: A critical review. Anal. Bioanal. Chem..

[55] Habibi G. (2013). Effect of drought stress and selenium spraying on photosynthesis and antioxidant activity of spring barley. Acta Agric. Slov..

[56] Saffaryazdi A., Lahouti M., Ganjeali A., Bayat H.J.N.S.B. (2012). Impact of selenium supplementation on growth and selenium accumulation on spinach (*Spinacia oleracea* L.). Plants.

[57] Van Hoewyk D. (2013). A tale of two toxicities: Malformed selenoproteins and oxidative stress both contribute to selenium stress in plants. Ann. Bot..

[58] Galasso C., Corinaldesi C., Sansone C. (2017). Carotenoids from Marine Organisms: Biological Functions and Industrial Applications. Antioxidants.

[59] Mostofa M.G., Hossain M.A., Siddiqui M.N., Fujita M., Tran L.S.P. (2017). Phenotypical, physiological and biochemical analyses provide insight into selenium-induced phytotoxicity in rice plants. Chemosphere.

[60] Seppänen M., Turakainen M., Hartikainen H. (2003). Selenium effects on oxidative stress in potato. Free Radic. Res..

[61] Wang J.M., Cappa J.J., Harris J.P., Edger P.P., Zhou W., Pires J.C., Adair M., Unruh S.A., Simmons M.P., Schiavon M. (2018). Transcriptome-wide comparison of selenium hyperaccumulator and nonaccumulator species provides new insight into key processes mediating the hyperaccumulation syndrome. Plant Biotechnol. J..

[62] Gouveia G.C.C., Galindo F.S., Lanza M.G.D.B., Silva A.C.D., Mateus M.P.D., da Silva M.S., Tavanti R.F.R., Tavanti T.R., Lavres J., dos Reis A.R. (2020). Selenium toxicity stress-induced phenotypical, biochemical and physiological responses in rice plants: Characterization of symptoms and plant metabolic adjustment. Ecotoxicol. Environ. Saf..

[63] Sun M.F., Hui X.R., Tong C.L., Yuan L.Y., Zhang D.J. (2022). The Physiological and Molecular Responses of Exogenous Selenium to Selenium Content and Fruit Quality in Walnut. Phyton-Int. J. Exp. Bot..

[64] Xu Y.M., Guo Y.K., Bai J.G., Shang L., Wang X.J. (2008). Effects of long-term chilling on ultrastructure and antioxidant activity in leaves of two cucumber cultivars under low light. Physiol. Plant.

[65] Feng R.W., Wei C.Y., Tu S.X. (2013). The roles of selenium in protecting plants against abiotic stresses. Environ. Exp. Bot..

[66] Zhao Y.G., Wu P.T., Wang Y.K., Feng H. (2013). Different approaches for selenium biofortification of pear-jujube (M. cv. Lizao) and associated effects on fruit quality. J. Food Agric. Environ..

[67] Van Hoewyk D., Takahashi H., Inoue E., Hess A., Tamaoki M., Pilon-Smits E.A.H. (2008). Transcriptome analyses give insights into selenium-stress responses and selenium tolerance mechanisms in Arabidopsis. Physiol. Plant.

[68] Li X., Wu Y., Li B., Yang Y., Yang Y. (2018). Selenium Accumulation Characteristics and Biofortification Potentiality in Turnip (Brassica rapa var. rapa) Supplied with Selenite or Selenate. Front. Plant Sci..

[69] Sors T.G., Ellis D.R., Salt D.E. (2005). Selenium uptake, translocation, assimilation and metabolic fate in plants. Photosynth. Res..

[70] Zhang L.H., Hu B., Li W., Che R.H., Deng K., Li H., Yu F.Y., Ling H.Q., Li Y.J., Chu C.C. (2014). OsPT2, a phosphate transporter, is involved in the active uptake of selenite in rice. New Phytol..

[71] Li H.F., McGrath S.P., Zhao F.-J. (2008). Selenium uptake, translocation and speciation in wheat supplied with selenate or selenite. New Phytol..

[72] Ríos J.J., Blasco B., Cervilla L.M., Rubio-Wilhelmi M.M., Ruiz J.M., Romero L. (2008). Regulation of sulphur assimilation in lettuce plants in the presence of selenium. Plant Growth Regul..

[73] Wu L.N., Jiang Y., Zhao F.Y., He X.F., Liu H.F., Yu K. (2020). Increased organic fertilizer application and reduced chemical fertilizer application affect the soil properties and bacterial communities of grape rhizosphere soil. Sci. Rep..

[74] Xu Y., Li Y. (2023). Effects of Sodium Selenite on the Rhizosphere Environment, Growth, and Physiological Traits of Oilseed Rape (*Brassica napus* L.). Agronomy.

[75] Jiao L., Cao X., Wang C., Chen F., Zou H., Yue L., Wang Z. (2023). Crosstalk between in situ root exudates and rhizobacteria to promote rice growth by selenium nanomaterials. Sci. Total Environ..

[76] Timmusk S., Abd El-Daim I.A., Copolovici L., Tanilas T., Kannaste A., Behers L., Nevo E., Seisenbaeva G., Stenstrom E., Niinemets U. (2014). Drought-Tolerance of Wheat Improved by Rhizosphere Bacteria from Harsh Environments: Enhanced Biomass Production and Reduced Emissions of Stress Volatiles. PLoS ONE.

[77] Lai C.-Y., Wen L.-L., Shi L.-D., Zhao K.-K., Wang Y.-Q., Yang X., Rittmann B.E., Zhou C., Tang Y., Zheng P. (2016). Selenate and Nitrate Bioreductions Using Methane as the Electron Donor in a Membrane Biofilm Reactor. Environ. Sci. Technol..

[78] Sultana N., Zhao J., Cai Y., Rahman G.K.M.M., Alam M.S., Faheem M., Ho A., Jia Z. (2022). Methanotrophy-driven accumulation of organic carbon in four paddy soils of Bangladesh. Pedosphere.

[79] Zhang M., Xing G.F., Tang S.H., Pang Y.W., Yi Q., Huang Q.Y., Huang X., Huang J.F., Li P., Fu H.T. (2019). Improving soil selenium availability as a strategy to promote selenium uptake by high-Se rice cultivar. Environ. Exp. Bot..

[80] Zhang M., Tang S.H., Zhong S.Z., Li P., Fu H.T. (2018). Effects of selenium fertilization on selenium availability in rice soil. Chin. J. Appl. Ecol..

[81] Alperin M.J., Hoehler T.M. (2009). Anaerobic Methane Oxidation by Archaea/Sulfate-Reducing Bacteria Aggregates: 1. Thermodynamic and Physical Constraints. Am. J. Sci..

[82] Chen J., Zhou Z.C., Gu J.D. (2014). Occurrence and diversity of nitrite-dependent anaerobic methane oxidation bacteria in the sediments of the South China Sea revealed by amplification of both 16S rRNA and genes. Appl. Microbiol. Biot..

[83] Legierse A., Struik Q., Smith G., Medrano M.J.E., Weideveld S., Van Dijk G., Smolders A.J.P., Jetten M., Veraart A.J., Welte C.U. (2023). Nitrate-dependent anaerobic methane oxidation (N-DAMO) as a bioremediation strategy for waters affected by agricultural runoff. FEMS Microbiol. Lett..

[84] Cui J., Li Y., Wang C., Kim K.S., Wang T., Liu S. (2018). Characteristics of the rhizosphere bacterial community across different cultivation years in saline-alkaline paddy soils of Songnen Plain of China. Can. J. Microbiol..

[85] Kumar U., Nayak A.K., Shahid M., Gupta V.V.S.R., Panneerselvam P., Mohanty S., Kaviraj M., Kumar A., Chatterjee D., Lal B. (2018). Continuous application of inorganic and organic fertilizers over 47 years in paddy soil alters the bacterial community structure and its influence on rice production. Agric. Ecosyst. Environ..

[86] Kravchenko I.K., Semenov V.M., Kuznetsova T.V., Bykova S.A., Dulov L.E., Pardini D., Gispert M., Boeckx P., Van Cleemput O., Gal’chenko V.F. (2005). Physicochemical and biological factors affecting atmospheric methane oxidation in gray forest soils. Microbiology.

