# Supplementary material for: Selenium-Induced Enhancement in Growth and Rhizosphere Soil Methane Oxidation of Prickly Pear
Source: Plants (Basel). 2024 Mar 7;13(6):749. doi: 10.3390/plants13060749 (PMC10974067; doi:10.3390/plants13060749)
Supplement: Supplementary file 1 [file plants-13-00749-s001.zip › plants-2866229-supplementary.pdf]

Supplementary Table S1 Basic soil properties

Traits	pH	Organic carbon (g/kg)	Total N (g/kg)	Total P (g/kg)	Total K (g/kg)	Alkali-dissolved N (mg/kg)	Available P (mg/kg)	Available K (mg/kg)	Se (mg/kg)
Values	5.65±0.07	9.26±0.06	1.13±0.05	0.40±0.04	24.05±1.12	202.24±5.31	0.80±0.05	271.78±6.06	0.01±0.002

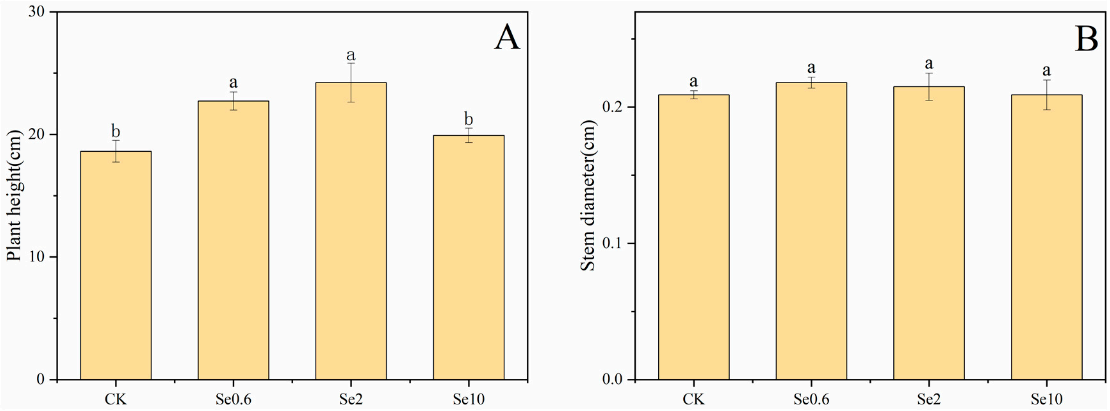

Supplementary Figure S1: Effect of soil selenium on height (A) and stem diameter (B) of prickly pear

Note: CK-0 mg/kg soil Se application; Se0.6-0.6 mg/kg soil Se application. Se2-2 mg/kg soil Se application. Se10-10 mg/kg soil Se application。 Different lowercase letters indicate significant differences between treatments and CK at 0.05 level.

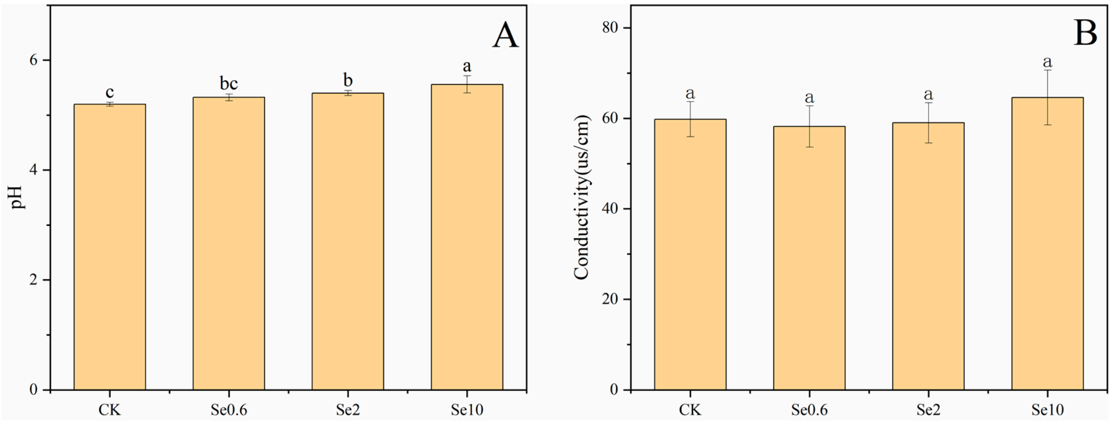

Supplementary Figure S2: Effect of different selenium on soil pH (A) and conductivity (B)

Note: CK-0 mg/kg soil Se application; Se0.6-0.6 mg/kg soil Se application. Se2-2 mg/kg soil Se application. Se10-10 mg/kg soil Se application. Different lowercase letters indicate significant differences between treatments and CK at 0.05 level.
